# The Relationship between Mitochondrial Reactive Oxygen Species Production and Mitochondrial Energetics in Rat Tissues with Different Contents of Reduced Coenzyme Q

**DOI:** 10.3390/antiox10040533

**Published:** 2021-03-29

**Authors:** Karolina Dominiak, Wieslawa Jarmuszkiewicz

**Affiliations:** Department of Bioenergetics, Adam Mickiewicz University, 61-614 Poznan, Poland; karolina.ogrodna@amu.edu.pl

**Keywords:** mitochondrial reactive oxygen species, coenzyme Q, mitochondrial energetics

## Abstract

We investigated the relationship between mitochondrial production of reactive oxygen species (ROS) and mitochondrial energetics in various rat tissues with different contents of the reduced coenzyme Q (Q) pool (Q9 + Q10). Our results indicate that similar to the tissue level, mitochondrial H_2_O_2_ release under nonphosphorylating conditions was strongly dependent on the amount of the reduced Q pool. Namely, in brain and lung mitochondria, less H_2_O_2_ release corresponded to a less reduced Q pool, while in liver and heart mitochondria, higher H_2_O_2_ release corresponded to a more reduced Q pool. We can conclude that the differences observed in rat tissues in the size of the reduced Q pool reflect different levels of ROS production and hence may reflect different demands for reduced Q as an antioxidant. Moreover, differences in mitochondrial H_2_O_2_ release were observed in different types of rat mitochondria during the oxidation of succinate (complex II substrate), malate plus glutamate (complex I substrate), and their mixture under phosphorylating and nonphosphorylating conditions. Our results indicate the existence of a tissue-specific maximum respiratory chain capacity in ROS production, possibly related to the membrane potential-mediated control of oxidative phosphorylation. We propose the use of a new parameter for the study of isolated mitochondria, RCR_ROS_, the ratio between the formation of mitochondrial ROS under nonphosphorylating and phosphorylating conditions, which represents the maximum factorial increase in mitochondrial ROS formation that can be achieved after all ADP is phosphorylated.

## 1. Introduction

Mitochondria are key organelles for cellular energy (ATP) production and reactive oxygen species (ROS) formation. The energy and ROS produced by mitochondria play an important role in various physiological and pathophysiological processes. Several sites that produce superoxide anion (O_2_^•−^) and/or hydrogen peroxide (H_2_O_2_) have been identified in mammalian mitochondria [1]. The predominant route of mitochondrial ROS (mROS) production by the electron transport chain is the premature leakage of electrons from complexes I, II, and III.

Coenzyme Q (Q) is a fat-soluble molecule present in all cell membranes, including the inner mito. hondrial membrane. Mitochondrial Q (mQ) plays a central role in the electron transport chain, transferring electrons between dehydrogenases and the oxidizing pathway (complex III and complex IV). Importantly, mQ is also involved in the formation of O_2_^•−^ from semiubiquinone radicals at the Q-binding sites of complexes I and III [1]. However, Q is not only involved in mROS production through the mitochondrial respiratory chain, but also an important antioxidant in mitochondria and the entire cell [2,3,4,5]. Reduced Q (ubiquinol) recycles other antioxidants such as vitamin E or vitamin C, and directly acts on free radicals or oxidants, reducing and neutralizing them. Reduced Q via binding free radicals inhibits lipid peroxidation and prevents oxidative modifications of DNA and proteins. Moreover, the Q redox state may be a useful marker of cellular oxidative stress [4].

The production of mROS depends on several factors, including the energetic status of the mitochondria, which affects the reduction of respiratory chain electron carriers [6,7,8,9]. Thus, mROS production is also dependent on the mitochondrial membrane potential (m∆Ψ), the major component of the proton electrochemical gradient that couples electron transport along the respiratory chain with ATP synthesis in the oxidative phosphorylation (OXPHOS) process. It should be emphasized that most studies on mROS production of isolated mitochondria concern nonphosphorylating (resting) conditions, which are accompanied by a large reduction of respiratory chain electron carriers and a high m∆Ψ. However, the change in mROS production that occurs as a result of mitochondrial transition from nonphosphorylating conditions (state 4 with high mROS production) to phosphorylating conditions (state 3 with lower m∆Ψ and lower mROS production) is of great physiological importance. This change demonstrates the extent of mROS production that accompanies mitochondrial function when the respiratory chain is uninhibited.

The aim of our work was to investigate the production of mROS in various rat tissues, i.e., in the heart and liver (tissues with high Q content) and in the brain and lungs (tissues with low Q content) under nonphosphorylating conditions (in the absence of ADP) and under phosphorylating conditions with active OXPHOS (in the presence of ADP). We studied mROS production in the context of cellular (tissue) and mitochondrial Q pool (mQ9 and mQ10) content, especially the content of the reduced Q pool. Moreover, the relationship between mROS formation and m∆Ψ was described for mitochondria isolated from the tested tissues.

## 2. Materials and Methods

### 2.1. Animals

The experiments were performed on adult 3- to 4-month-old male Wistar rats weighing 350–450 g. The animals were bred in the animal house at the AMU Advanced Technology Center, Poznan, Poland. They were given free access to water and pellet food and were housed under standard humidity and temperature conditions on a 12 h light/dark cycle. Experimental protocols involving animals, their surgery, and care were in compliance with the guidelines of the European Community Council Directive on the protection of animals used for scientific purposes. Animals were sacrificed by decapitation, and all efforts were made to minimize suffering. As no procedures were performed using live animals and as they were sacrificed for scientific purposes, no approval was needed for our study according to the Polish Animal Welfare Act.

### 2.2. Tissue Preparation and Mitochondria Isolation

All procedures were performed at 4 °C. To simultaneously isolate functional and intact mitochondria from rat heart, liver, brain, and lungs, the tissues were collected from one rat, placed in isolation medium A (pH 7.2) containing 50 mM Tris-HCl, 100 mM sucrose, and 0.5 mM ethylenediaminetetraacetic acid (EDTA) and washed several times. After removal of the connecting tissue and large vessels, the tissues were cut into small pieces over ice. The minced tissues were filtered through a strainer to remove the remaining blood and connecting tissue and then homogenized in isolation medium B containing 50 mM Tris-HCl (pH 7.2), 100 mM sucrose, 100 mM KCl, 1 mM KH_2_PO_4_, 0.1 mM ethylene glycol-bis(β-aminoethyl ether)-*N*,*N*,*N*′,*N*′-tetraacetic acid (EGTA), and 0.5 mM EDTA using a Teflon or glass pestle. The strained homogenates were centrifuged at 900× *g* for 10 min. The resultant supernatants were filtered. Part of the filtered homogenates was used for studies (measurements of H_2_O_2_ release and the Q content and reduction level). The greater part of the homogenates was supplemented with isolation medium B with 0.2% BSA and centrifuged at 17,800× *g* for 10 min. Mitochondrial pellets suspended in isolation medium B without BSA were centrifuged at 900× *g* for 8 min. The supernatants were filtered and again centrifuged at 17,800× *g* for 10 min. The final pellets of mitochondria were then resuspended in medium C containing 10 mM Tris-HCl, pH 7.2, 75 mM sucrose, and 225 mM mannitol. The protein concentrations of homogenates and mitochondria were determined by the Bradford method.

All functional measurements were performed on freshly isolated mitochondria at 35 °C. Only mitochondria with good integrity of the mitochondrial outer membrane (97–100%) (measured as described in [10]) were used for the study.

### 2.3. Measurements of the Tissue and Mitochondrial Q Content

Concentrations of tissue and mitochondrial Q9 (a dominant Q form in rat tissues) and Q10 (a less abundant Q form in rat tissues) were determined in homogenates and mitochondria by an extraction technique and HPLC detection [9,11]. Both reduced (275 nm) and oxidized (290 nm) forms of Q9 and Q10 were detected. For the quantification and calibration of the Q9 and Q10 peaks, commercial Q9 and Q10 were used. The total and reduced Q9 and Q10 pools were determined in rat tissues and mitochondria under fully oxidizing conditions, i.e., in the absence of respiratory Q-reducing substrates. Before Q extraction, homogenates (7 mg) and mitochondria (3 mg) were incubated with gentle agitation for 20 min in 3 mL of standard assay medium, which comprised 75 mM sucrose, 225 mM mannitol, 10 mM KCl, 5 mM KH_2_PO4, 0.5 mM EDTA, 10 mM Tris/HCl (pH 7.2), and 0.2% BSA.

### 2.4. Mitochondrial Membrane Potential Measurements in Isolated Mitochondria

mΔΨ was measured using a tetraphenyl-phosphonium (TPP+)-specific electrode [12,13] in 3 mL of standard assay medium with 3 mg of mitochondrial protein (1 mg × mL^−1^). The TPP+-electrode was calibrated based on three sequential additions (1.6, 1.6, and 3.2 µM) of TPP+. After each run, 0.5 µM FCCP was added to release TPP+ to correct the baseline. To calculate the mΔΨ value, the volume of rat mitochondrial matrix was assumed to be 2.0 μL × mg^−1^ protein.

ADP (170 μM) was added to determine the mΔΨ of phosphorylating (state 3) respiration. The mΔΨ of nonphosphorylating (resting state, State 4) respiration was determined when ADP was exhausted or in measurements without exogenous ADP. The respiratory substrates were as follows: 5 mM succinate, 5 mM malate and 5 mM glutamate, and a mixture of 5 mM succinate, 5 mM malate, and 5 mM glutamate.

### 2.5. Assay of H_2_O_2_ Release

The H_2_O_2_ release rate was measured by the Amplex Red assay [9,14]. Horseradish peroxidase (0.14 U × mL^−1^) catalyzes the H_2_O_2_-dependent oxidation of nonfluorescent Amplex Red (5 μM) to fluorescent resorufin red. The release of O_2_^•−^ was also captured by the addition of exogenous superoxide dismutase (SOD, 5U × mL^−1^) to convert O_2_^•−^ to H_2_O_2_ in the assay medium. The fluorescence kinetics were followed for 40 min at 545 nm/590 nm using a Tecan multimode reader (Infinite M200 PRO) with 24-well plates. Homogenates (0.2 mg of protein) were incubated in 0.5 mL of standard assay medium with a mixture of 5 mM succinate, 5 mM malate, and 5 mM glutamate in the absence (nonphosphorylating conditions) or presence of 0.75 mM ADP (phosphorylating conditions). Mitochondria (0.26 mg of protein) were incubated in 0.5 mL of the standard incubation medium with 5 mM succinate, 5 mM malate, and 5 mM glutamate or a mixture of 5 mM succinate, 5 mM malate, and 5 mM glutamate in the absence (nonphosphorylating state 4 conditions) or presence of 1.5 mM ADP (phosphorylating state 3 conditions). H_2_O_2_ release of nonphosphorylating (resting state, state 4) respiration was determined after ADP depletion or in measurements without exogenous ADP. Reactions were calibrated with known amounts of H_2_O_2_.

Limitations resulting from the technique used to measure H_2_O_2_ release should be considered. The Amplex Red Assay does not take into account the portion of H_2_O_2_ produced in the mitochondrial matrix that is removed by matrix peroxidases. Furthermore, it is important to consider the limited diffusion of H_2_O_2_ from the matrix outside the mitochondria in living cells [15]. Amplex Red is readily, independently of H_2_O_2_, converted into fluorescent resorufin by a carboxylesterase. Therefore, it would be worth carrying out measurements in the presence of phenylmethylsulfonyl fluoride, which inhibits this process [16].

### 2.6. Statistical Analysis

The results are presented as the means ± SD obtained from at least 4 independent homogenate preparations or mitochondrial isolations, in which each determination was performed at least in duplicate. Each isolation of lung, brain, liver, and heart mitochondria was performed from one animal. Significant differences were determined via unpaired *t*-tests or ANOVAs (followed by Tukey’s post hoc comparisons for *p* < 0.05 from an ANOVA). Differences were considered to be statistically significant if *p* < 0.05 (*), *p* < 0.01 (**), or *p* < 0.001 (***).

## 3. Results

### 3.1. Distribution of Q9, Q10 and total Q (Q9 + Q10) in the Cells and Mitochondria of Different Rat Tissues

Four rat tissues were selected for the study, i.e., heart, liver, brain, and lungs, with different Q contents (Table 1). Our determinations of the total (cellular) amount of Q9, Q10 and total of Q9 + Q10 confirmed previous studies [5,17], indicating that in different rat tissues the level of both Q homologs varies, with Q9 being the dominant form. In 3- to 4-month-old male rats, the total amount of tissue Q content (Q9 + Q10) varied ~7-fold, with the highest in the heart and the lowest in the lungs (Table 1). Similarly, in the examined tissues, the total amount of mitochondrial Q content (Q9 + Q10) varied ~7-fold, again with the highest in the heart and the lowest in the lungs. Q9 was also the dominant form in mitochondria. The proportion between Q9 and Q10 varied between tissues and their mitochondria.

We also determined the levels of both oxidized and reduced Q pools of Q9 and Q10 in tissues and mitochondria (Table 2), as the reduced Q is the form that exerts an antioxidant effect. The percentage of reduced Q9 and Q10 also differed in different tissues. The highest reduction of both forms of Q was observed in the liver, both at the tissue and mitochondrial levels. Namely, in the liver ~90% of the Q9 and Q10 were reduced in tissues, as was Q10 in the mitochondria. Only mitochondrial Q9 showed a smaller but still high reduction (~50%) in liver mitochondria. In the heart, the level of reduction of both Qs was similar, i.e., ~25–29% in tissues and ~15–19% in mitochondria. Interestingly, almost the entire pool of reduced Q in the lungs consisted of Q10, both at the tissue and mitochondrial levels. The lowest level of reduction of both Qs was observed in the brain. No reduced Q pool was detected at the tissue level and was ~9% in mitochondria (for both Q9 and Q10). The differences in the size of the reduced Q pools in different tissues indicate different needs for reduced Qs as specific antioxidants at both the tissue and mitochondrial levels.

### 3.2. The Relationship between H_2_O_2_ Formation and the Content of the Total and Reduced Q Pool (Q9 + Q10) in Rat Tissue Homogenates

**Figure 1:**
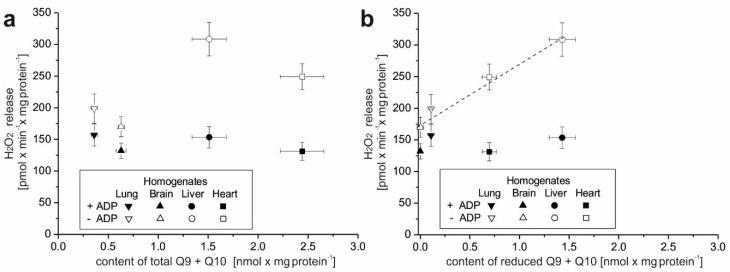
H_2_O_2_ release versus the content of total (**a**) and reduced (**b**) Q pools (Q9 + Q10) in rat tissue homogenates. H_2_O_2_ release measurements were performed in the absence or presence of ADP with succinate and malate plus glutamate as mitochondrial respiratory substrates. The total and reduced Q9 + Q10 pools were measured under fully oxidizing conditions (no respiratory Q-reducing substrates). Mean ± SD. (**b**) The linear regression between H_2_O_2_ release in the absence of ADP and the content of reduced Q9 + Q10 is shown (r = 0.988, *p* = 0.01).

We then measured the level of H_2_O_2_ release in tissue homogenates under mitochondrial respiratory chain activation conditions by adding succinate (substrate for complex II) and malate plus glutamate (substrate for complex I). The measurement was performed under OXPHOS activation conditions (in the presence of ADP) and in the absence of ADP. The obtained results were presented for the total (Figure 1a) and reduced (Figure 1b) Q (Q9 + Q10) pools.

Interestingly, in all four examined tissues a similar release of H_2_O_2_ (~130–155 pmol × min^−1^ × mg protein^−1^) was observed under active OXPHOS conditions (Figure 1a,b). In the absence of ADP, the release of H_2_O_2_ was higher and was ~170–200 pmol × min^−1^ × mg protein^−1^ for the brain and lungs, ~250 pmol × min^−1^ × mg protein^−1^ for the heart, and ~310 pmol × min^−1^ × mg protein^−1^ for the liver. These results indicate a greater ROS production capacity in the heart and liver tissues (the highest in the liver) than in the lung and brain tissues.

The increased H_2_O_2_ release (in the absence of ADP) in the heart and liver was accompanied by a much larger total Q (Q9 + Q10) pool than that in the lung and brain (Figure 1a). When comparing the release of H_2_O_2_ with the reduced Q pool (Figure 1b), a linear relationship was observed in the absence of ADP. Under these conditions, less H_2_O_2_ release corresponded to a less reduced Q pool and higher H_2_O_2_ release corresponded to a more reduced Q pool. Overall, our results indicate that rat tissues that can produce more ROS have larger total and reduced Q pools (Q9 + Q10) and that tissue ROS production seems to be proportional to the reduced tissue Q pool.

### 3.3. Comparison of H_2_O_2_ Production in Mitochondria Isolated from Various Rat Tissues during the Oxidation of Succinate (Substrate for Complex II) and Malate Plus Glutamate (Substrate for Complex I)

**Figure 2:**
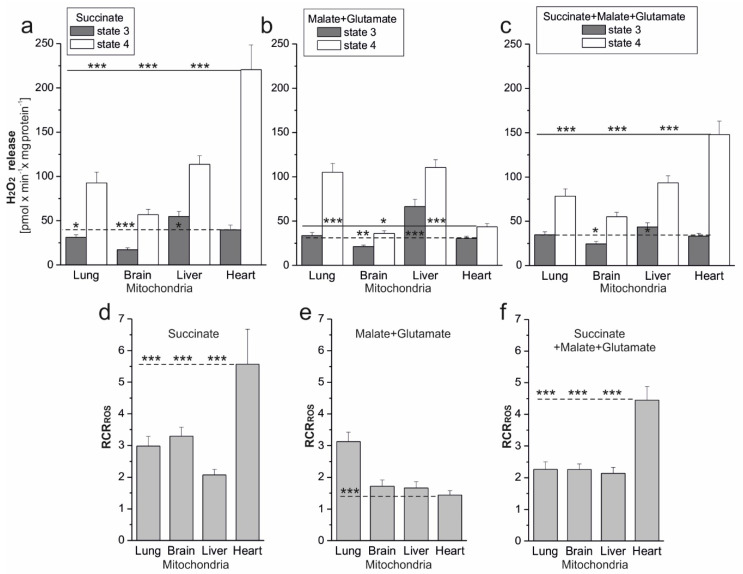
H_2_O_2_ release in isolated rat mitochondria oxidizing succinate (**a**), malate plus glutamate (**b**) and a mixture of succinate and malate plus glutamate (**c**) under phosphorylating (state 3) and nonphosphorylating (state 4) conditions. RCR_ROS_, respiratory control ratio of mROS formation (state 4 H_2_O_2_ formation/state 3 H_2_O_2_ formation) with various respiratory substrates (**d**–**f**). Mean ± SD; *n* = 5. *p* < 0.05 (*), *p* < 0.01 (**), or *p* < 0.001 (***), comparison vs. mean values for heart mitochondria.

The release of H_2_O_2_ in mitochondria isolated from different rat tissues was then compared. Mitochondrial H_2_O_2_ release was measured under the phosphorylating (state 4) and nonphosphorylating (state 4) conditions with succinate (substrate of complex II), malate, and glutamate (substrate of complex I) and a mixture of succinate and malate plus glutamate (two active electron inputs to the respiratory chain) (Figure 2).

For all tissues and combinations of respiratory substrates used, H_2_O_2_ release in state 3 was significantly lower than that in state 4. Figure 2d–f show the calculated ratio of H_2_O_2_ release in state 4, i.e., under conditions of highest ROS production when OXPHOS is inactive, to H_2_O_2_ release in state 3, i.e., when the respiratory chain is at maximum respiratory activity. We propose to call this ratio the respiratory control ratio of ROS formation (RCR_ROS_). The ratio represents the maximum factorial increase in mitochondrial ROS formation that can be achieved after all ADP is phosphorylated. In the case of the examined mitochondria, the highest RCR_ROS_ values were observed in the heart mitochondria during the oxidation of succinate (~5.5) (Figure 2d) and the mixture of complexes I and II substrates (~4.5) (Figure 2f). In the case of malate plus glutamate oxidation, the greatest increase in H_2_O_2_ release after ADP depletion was observed for lung mitochondria (~3.2) (Figure 2e).

Some properties can be observed when comparing H_2_O_2_ release in mitochondria from the examined rat tissues. Namely, regardless of the type of respiratory substrate, H_2_O_2_ release was the lowest in the brain mitochondria for both respiratory states. On the other hand, the highest H_2_O_2_ release was observed for succinate and a mixture of complex I and II substrates in heart mitochondria. In the case of lung and liver mitochondria, H_2_O_2_ release was similar for complex I substrate oxidation and for complex II substrate oxidation under both respiratory conditions. In the case of heart and brain mitochondria, H_2_O_2_ release under nonphosphorylating conditions was significantly higher during the oxidation of succinate (substrate for complex II) than during the oxidation of malate plus glutamate (substrate for complex I). Interestingly, for all types of mitochondria, in both respiratory states, the H_2_O_2_ release observed at the involvement of both electron inputs on the respiratory chain (oxidation of complex I and II substrates) did not exceed the H_2_O_2_ release observed with a single best substrate entry. This observation indicates the existence of a maximum respiratory chain capacity in ROS production, i.e., an upper limit in state 4 and a lower limit in state 3.

### 3.4. The Relationship between Mitochondrial H_2_O_2_ Formation and the Content of the Reduced mQ Pool (mQ9 + mQ10) and m∆Ψ

**Figure 3:**
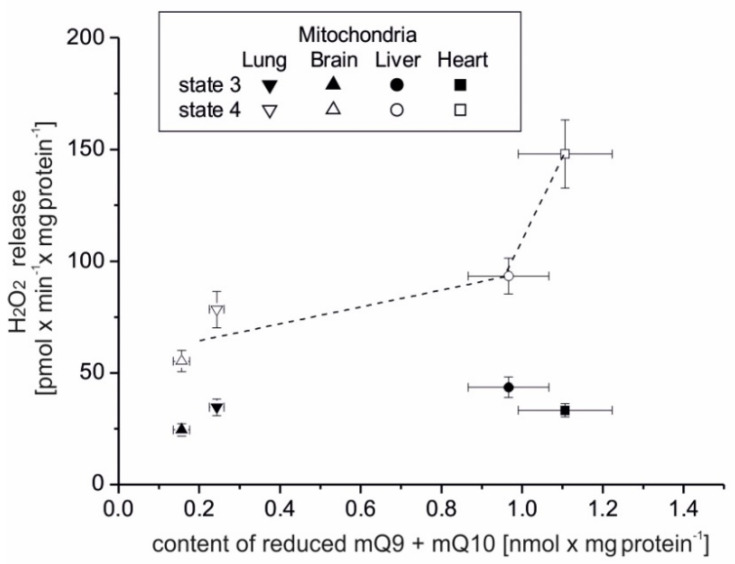
H_2_O_2_ release and the content of the reduced mQ pool (mQ9 + mQ10) in rat mitochondria isolated from various tissues. H_2_O_2_ formation measurements were performed in the absence (state 4) or presence (state 3) of ADP with a mixture of succinate and malate plus glutamate. The reduced mQ9 + mQ10 pools were measured under fully oxidizing conditions (no respiratory Q-reducing substrates). Mean ± SD; *n* = 5.

Figure 3 presents the relationship between H_2_O_2_ release and the content of the reduced mQ pool (mQ9 + mQ10) in various rat mitochondria. H_2_O_2_ formation was measured with a mixture of succinate and malate plus glutamate (complex II and complex I substrates) under phosphorylating (state 3) and nonphosphorylating (state 4) conditions. The reduced mQ9 + mQ10 pools were measured under fully oxidizing conditions (no respiratory Q-reducing substrates) (Table 2). As at the tissue level (Figure 1), H_2_O_2_ formation in mitochondria respiring in state 3 was not strongly associated with the amount of reduced Q pool (Figure 3). However, such a relationship (although not linear) can be observed in the case of mitochondria, which oxidize substrates under nonphosphorylating conditions. Under these conditions, in the brain and lung mitochondria, less H_2_O_2_ release corresponded to a less reduced mQ pool and in the liver and heart mitochondria, higher H_2_O_2_ release corresponded to a more reduced mQ pool. This relationship was much steeper for the amount of reduced mQ above 1 nmol× min^−1^ × mg protein^−1^.

**Figure 4:**
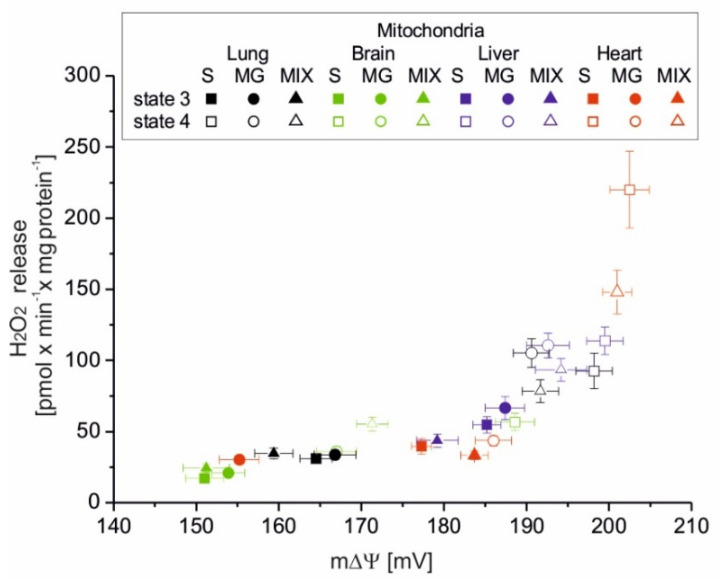
The relationship between H_2_O_2_ release and m∆Ψ in isolated rat mitochondria oxidizing succinate (S), malate plus glutamate (MG), and a mixture of succinate and malate plus glutamate (MIX) under phosphorylating (state 3) and nonphosphorylating (state 4) conditions. Mean ± SD; *n* = 5.

Figure 4 shows the relationships between H_2_O_2_ formation versus m∆Ψ in various rat mitochondria oxidizing succinate, malate plus glutamate, and a mixture of succinate and malate plus glutamate under phosphorylating (state 3) and nonphosphorylating (state 4) conditions. The points obtained for all tested mitochondria and respiratory substrates form a single relationship clearly showing the nonlinear dependence of mROS formation on m∆Ψ. Up to ~180 mV, this relationship is not steep, with the slope clearly increasing between 180 and 200 mV. There is a threshold mΔΨ value (~200 mV) above which even a small increase in mΔΨ gives rise to a large stimulation of H_2_O_2_ release by mitochondria. The steepest relationship between H_2_O_2_ release and m∆Ψ occurs for heart mitochondria, while for brain mitochondria this relationship is much less steep.

## 4. Discussion

The present study focuses on the relationship between mROS generation and mitochondrial energetics in various rat tissues with different reduced Q pool contents. First, we found that rat tissues at the tissue and mitochondrial levels differ not only in the content of total Q pools (Q9, Q10, and Q9 + Q10) but also in the content of reduced Q pools (Q9, Q10, and Q9 + Q10). At the tissue level, we found a similar percentage reduction in the total Q9 + Q10 pool previously observed in various rat tissues [5]. However, reduced Q pools for the mitochondria of various rat tissues as well as the relationship of the total and reduced Q pools at the tissue and mitochondrial levels to ROS production have not been studied. Our results indicate that rat tissues that can produce more ROS (liver and heart) in the absence of ADP have larger total and reduced Q pools (Q9 + Q10) than tissues producing less ROS (lung and brain). At the tissue level, ROS production seems to be proportional to the reduced tissue Q pool (Q9 + Q10). Similar to the tissue level, H_2_O_2_ release in mitochondria respiring under nonphosphorylating conditions (state 4) was strongly dependent (although nonlinearly) on the amount of reduced Q pool. Namely, in the brain and lung mitochondria less H_2_O_2_ release corresponded to a less reduced mQ pool, while in the liver and heart mitochondria, higher H_2_O_2_ release corresponded to a more reduced mQ pool. Thus, the observed differences in the size of the reduced Q pool (Q9 + Q10) reflect different levels of ROS production and hence may reflect different demands for reduced Qs as antioxidants at both the tissue and mitochondrial levels. It is well accepted that in their reduced form Qs have antioxidant activity, as scavengers of ROS or lipid radicals and regenerators of α-tocopherol from the α-tocopheroxyl radical [3,4,5]. Obviously, at the tissue level, the larger total and reduced Q pools are also related to the number of mitochondria in a given tissue. On the other hand, at the mitochondrial level, when the content of reduced mQ pool and mROS production are presented per mg of mitochondrial protein, increased production of mROS under nonphosphorylating conditions, combined with an increased reduced mQ pool, indicates a greater need for this antioxidant. However, further research, including measuring redox balance and other main antioxidant molecules such as glutathione, is necessary to elucidate the phenomenon described in this work. Our results also indicate that the Q redox state in a given tissue may be a useful marker of cellular oxidative stress. However, it must be remembered that Q levels decline with age in human and rat tissues [17]; hence, the levels of reduced Qs may also change.

Of great physiological importance is the change in mROS formation, which occurs during the transition of mitochondria from nonphosphorylating conditions (state 4, with increased mROS production) to phosphorylation conditions (state 3, with decreased mROS production). This change demonstrates the extent of mROS production that accompanies mitochondrial function when the respiratory chain is uninhibited. The parameter proposed by us, i.e., RCR_ROS_, the ratio between mROS formation in state 4 versus mROS formation in state 3, represents the maximum factorial increase in mROS formation that can be achieved after the end of ADP phosphorylation. The RCR_ROS_ can be determined for isolated mitochondria. In vivo, mitochondria can shift rapidly between these conditions inducing a change in m∆Ψ and thereby in mROS formation. In the present study, during nonphosphorylating respiration, high mΔΨ values were accompanied by increased mROS formation in tested rat mitochondria. In contrast, during phosphorylating respiration, the relative decrease in mΔΨ was accompanied by decreased mROS formation. It has been shown previously that in nonphosphorylating rat heart mitochondria, mROS generation strongly but nonlinearly depends upon mΔΨ, increasing at mΔΨ greater than that of state 3 [18]. Our results obtained with heart mitochondria confirm this observation. In addition, we have shown that the measurements of H_2_O_2_ release and mΔΨ obtained for four types of rat mitochondria and different respiratory substrates form one relationship clearly showing the nonlinear dependence of mROS production on mΔΨ. However, it should be remembered that mROS production is not a direct function of mΔΨ under the conditions of inhibition of the Q-oxidizing segment of the respiratory chain (complex III or complex IV) [9].

To date, at least 11 sites that produce O_2_^•−^ and/or H_2_O_2_ have been identified in mammalian mitochondria [1,19]. The contributions of specific sites of the mitochondrial respiratory chain to the production of ROS in mitochondria depend very strongly on the substrates being oxidized [20]. Under our experimental conditions, during succinate oxidation, the flavin site of complex II (site II_F_) and the Q_o_ and Q_i_ sites of complex III (site III_Qo_ and site III_Qi_) participated in mROS production. Moreover, involvement of the flavin (IF site) and the Q site (IQ site) of complex I due to reverse electron transport cannot be ruled out. When the malate was oxidized, all mROS production sites mentioned were active. The purpose of our research was not to determine the contribution of individual mROS production sites but to determine the total mROS production when electrons enter the respiratory chain via complex I and/or complex II under phosphorylating (state 3) and nonphosphorylating (state 4) conditions for the mitochondria of various rat tissues. As far as we know, there has been no such research before. Comparing mROS production in rat lung, brain, liver, and heart mitochondria, it can be seen that, regardless of the substrate used under phosphorylating conditions, H_2_O_2_ release was lowest in brain mitochondria and highest in liver mitochondria. Under nonphosphorylating conditions, H_2_O_2_ release was lowest in brain mitochondria (regardless of the substrate used) and highest in heart mitochondria (during the oxidation of succinate alone or in combination with malate plus glutamate). In the case of liver and lung mitochondria, no major quantitative differences in mROS production can be seen during the oxidation of the substrates of complex I and complex II. In the case of the heart and brain mitochondria, H_2_O_2_ release under nonphosphorylating conditions was significantly higher when complex II was involved compared to complex I. Interestingly, for all types of mitochondria, in both respiratory states, the H_2_O_2_ release observed at the involvement of both complex I and complex II did not exceed the H_2_O_2_ release observed with a single best substrate entry. This observation indicates the existence of a maximum respiratory chain capacity in ROS production, i.e., an upper limit in state 4 and a lower limit in state 3, possibly related to the OXPHOS control mediated by mΔΨ. Measurements of mROS formation under physiological conditions in the absence of electron transport inhibitors may be helpful in assessing the overall intrinsic production of mROS in mitochondria and, in the longer term, the physiological role of these signaling molecules in mitochondrial dysfunction.

Comparing mitochondria isolated from different tissues is difficult due to possible different contaminants. Therefore, the data obtained should be interpreted with caution. In the case of liver mitochondria, the obtained results of the measurement of H_2_O_2_ release may be underestimated due to contamination with peroxisomal catalase. On the other hand, in the case of brain mitochondria, the data (H_2_O_2_ release, amount of mQ pool, and mΔΨ) may be underestimated due to contamination with synaptosomes and myelin. Moreover, when interpreting the obtained results, one should take into account the limitations resulting from the applied technique of measuring H_2_O_2_ release, mentioned in Materials and Methods.

## 5. Conclusions

We studied the change in mROS production that occurs as a result of transition from nonphosphorylating conditions to phosphorylating conditions in mitochondria of various rat tissues with different contents of the reduced Q. This change, described by the RCR_ROS_ parameter, shows the extent of mROS production that accompanies mitochondrial function when the respiratory chain is uninhibited. We found that ROS production under nonphosphorylating conditions was strongly dependent on the amount of reduced Q and may reflect different requirements for reduced Q as an antioxidant.

## Figures and Tables

**Table 1:** Q9, Q10, and total Q (Q9 + Q10) content in different rat tissues and their mitochondria.

Rat Tissue	Tissue Content (nmol mg^−1^ Protein)			Mitochondrial Content (nmol mg^−1^ Protein)		
	Q9	Q10	Q9 + Q10	mQ9	mQ10	mQ9 + mQ10
Heart	2.32 ± 0.26	0.12 ± 0.01	2.44 ± 0.25	5.28 ± 0.66	0.54 ± 0.07	5.82 ± 0.74
Liver	1.41 ± 0.13	0.10 ± 0.01	1.51 ± 0.17	1.59 ± 0.18	0.19 ± 0.02	1.78 ± 0.19
Brain	0.48 ± 0.05	0.15 ± 0.01	0.63 ± 0.05	1.36 ± 0.14	0.27 ± 0.03	1.63 ± 0.19
Lung	0.25± 0.03	0.11 ± 0.01	0.36 ± 0.04	0.64 ± 0.07	0.20 ± 0.02	0.84 ± 0.09 ^1^

^1^ Mean ± SD, *n* = 4–6.

**Table 2:** Percentage of the reduced Q9 and Q10 pools in rat tissues and mitochondria under fully oxidizing conditions (no respiratory Q-reducing substrates).

Rat Tissue	Tissue % of Reduced Q Pool			Mitochondria % of Reduced Q Pool		
	Q9	Q10	Q9 + Q10	mQ9	mQ10	mQ9 + mQ10
Heart	28.7 ± 2.6	25.3 ± 2.9	28.5 ± 3.0	18.9 ± 2.0	20.1 ± 2.1	19.0 ± 2.1
Liver	95.1 ± 9.7	87.4 ± 9.1	92.1 ± 9.5	49.7 ± 5.2	93.5 ± 9.9	68.6 ± 7.2
Brain	nd (0)	nd (0)	0	9.3 ± 1.1	11.1 ± 1.5	9.6 ± 1.2
Lung	nd (0)	100	42	6.5 ± 0.7	100	28.9 ± 2.2 ^1^

^1^ Mean ± SD, *n* = 4. nd, not detectable.

## Data Availability

The data presented in this study are openly available in Mendeley Data, V1 at doi:10.17632/sjtjy59ykv.1.
